# Sleep Studies for Clinical Indications during the First Year of Life: Infants Are Not Small Children

**DOI:** 10.3390/children9040523

**Published:** 2022-04-07

**Authors:** Athanasios Kaditis, David Gozal

**Affiliations:** 1 Division of Pediatric Pulmonology, First Department of Pediatrics, National and Kapodistrian University of Athens School of Medicine, 115 27 Athens, Greece; 2 Cystic Fibrosis Department, Agia Sofia Children’s Hospital, 115 27 Athens, Greece; 3 Division of Pediatric Pulmonology, Pediatric Sleep Center, Department of Child Health, University of Missouri School of Medicine and MUHC Children’s Hospital, Columbia, MO 65201, USA; gozald@health.missouri.edu

## 1. Introduction

In a previous issue of *Children*, Guyon et al., presented longitudinal data on the sleep structure and maturation of spontaneous arousals in a limited number of preterm and term infants from the Autonomic Baby evaluation study [1]. Participants were evaluated after birth in the maternity unit and at 6 months of age at home, using 24 h polysomnography (PSG). Over the first 6 months of life and in agreement to what has been reported in previous published studies, diurnal total sleep time and proportion of active sleep decreased, whereas nocturnal sleep time and proportion of quiet sleep increased. For both preterm and term infants, arousals and especially those in active sleep, increased during nighttime sleep and decreased during daytime sleep. However, preterm infants had a lower total arousal index during diurnal and nocturnal sleep compared to term infants, potentially exposing them to a greater risk of SIDS.

Recording of spontaneous arousals is only one aspect of the ample information provided by PSG in infants. The bidirectional interactions between sleep and breathing are also reflected in the recordings. Even healthy young infants are predisposed to upper airway obstruction and hypoxemia both during sleep and wakefulness as a result of increased upper airway and chest wall compliance and immature control of breathing [2]. Chandrasekar et al., have recently reviewed the etiology, clinical manifestations, and treatment of obstructive sleep apnea syndrome (OSAS) in neonates, and have identified an obstructive apnea–hypopnea index (AHI) > 1 episode/h in PSG as the cut-off value for OSAS diagnosis [3]. However, this OSAS diagnostic threshold value of obstructive AHI has been a matter of discussion and contention in everyday clinical practice.

## 2. Maturational Changes of Sleep and Breathing during Infancy

In contrast to children and adolescents who have long periods of nocturnal continuous sleep, neonates manifest fragmented sleep periods that progressively consolidate to nighttime sleep with two daytime naps by late infancy [4]. Ultradian sleep cycles in neonates last 30–70 min with predominantly REM sleep onset, contrary to older infants and children who have cycles of 75–90 min duration with NREM sleep onset [5]. Sleep architecture evolves as chronological age progresses with full-term neonates spending 50% of total sleep time in REM (active) sleep that decreases gradually as infant sleep consolidates and its total duration declines [6]. Arousals facilitate sleep stage changes or transition to wakefulness and they also represent a physiologic protective response to airway obstruction and hypoxemia, the latter being a relatively weak arousing stimulus [5,7]. For this reason, scoring of episodes with decreased airflow accompanied by arousal is important for clinical purposes. Potentially as important would be events associated with reduced airflow not accompanied by arousal.

Respiratory parameters such as breathing frequency and its variability are affected by the sleep–wake state [8]. In full-term infants and from birth to 3 months of age, respiratory rate and its variability decrease linearly and stabilize from 4 to 6 months having their highest values during wakefulness, lowest over quiet sleep, and intermediate during active sleep [8]. Similar reductions in obstructive apnea index, hypopnea index, and desaturation (≥3%) index have been noted from 0 to 3 months of age, even in infants residing at high altitude [9,10,11].

REM (active) sleep represents a vulnerable period for respiration in the infant due to several reasons: (i) REM sleep is characterized by irregular respiratory rate and tidal volume in contrast to the regular breathing pattern noted during NREM or quiet sleep; (ii) the ventilatory responses of the central nervous system to both hypoxia and hypercapnia are reduced compared to those in NREM sleep; (iii) intercostal muscle activity diminishes leading to paradoxical chest and abdominal wall movements that are present for almost 100% of active sleep time in the newborn and for 20% at the age of 2 years; and (iv) functional residual capacity decreases in active sleep [12,13,14,15]. In addition, prevalence of periodic breathing in term infants is increased in REM sleep [16]. Therefore, the various stages of sleep–wake states should be taken under consideration when studying respiratory parameters in young infants.

## 3. Polysomnography and Polygraphy as Diagnostic Tools in Infancy

The term “infant sleep apnea” covers a wide range of events involving reduction or complete cessation of airflow (hypopnea or apnea, respectively) which may or may not be of pathological importance. The presence of respiratory effort signifies some degree of airway obstruction as the underlying cause of the event (obstructive hypopnea or apnea), while the absence of respiratory effort may or may not be related to inadequate central respiratory drive (central hypopnea or apnea). For example, *prolonged expiratory apnea* and particularly *post-sigh central apnea* are benign types of airflow cessation that may be misinterpreted as pathological central events [17,18]. Central apneas in patients with neuromuscular disease preceded by paradoxical breathing probably are due to the inability of weak respiratory muscles to move the chest wall and create airflow against an obstructed upper airway and for this reason are usually characterized as *“pseudo-central” apneas* [19]. Morbid sequelae of OSAS have been summarized in a recent review published in *Children* [20].

PSG is the gold standard method for evaluating apneas and hypopneas [21]. Nevertheless, its interpretation is problematic in infants and especially those younger than 6 months:

(i) Reference values for PSG, polygraphy, or nocturnal oximetry parameters in healthy infants have been reported in very few studies with limited numbers of participants [9,10,11,22,23]. Several studies including infants were published between 1981 and 2001, but scoring of tracings was not conducted using a consensus of guidelines such as the American Academy of Sleep Medicine (AASM) scoring rules, and no hypopneas were scored [24].
(ii) Although respiratory parameters in infants are affected by the specific state of sleep and/or wakefulness as was discussed above, no information is provided in these publications in terms of the specific proportions of sleep stages in the studied subjects. Thus, it is unknown whether sleep duration and architecture of infants undergoing PSG in clinical practice is directly comparable to that of healthy subjects in whom reference respiratory parameters were calculated.

In contrast, PSG interpretation is more straightforward in toddlers, children, and adolescents for several reasons:

(i) Subjects older than 1 year, and especially those older than 2 years, have more consolidated nocturnal sleep than infants, whose sleep is mostly fragmented and dispersed both during day and night.
(ii) Upper airway obstruction in children older than 2 years is usually prominent while asleep, but it is not so apparent during wakefulness. This phenomenon stems from the fact that older children have larger airway cross-sectional area, more mature control of breathing, and more efficient pharyngeal muscle tone than infants. Furthermore, severe upper airway obstruction that is caused by congenital disorders such as craniofacial abnormalities or laryngomalacia has been frequently managed at early young age.

In an effort to facilitate interpretation of PSGs in early life, appropriately adjusted rules for the scoring of sleep stages (wakefulness, NREM, REM and Transitional) in 0–2-month-old full-term infants have been developed and included in the AASM Manual for the Scoring of Sleep and Associated Events and a Task Force have provided recommendations on the visual scoring of sleep and arousals in infants [25,26]. In terms of respiratory events, there are special provisions in the AASM manual for the scoring of central apneas accompanied by bradycardia. However, some special characteristics of sleep-disordered breathing in infants are not addressed adequately:

(i) Classification of hypopneas to obstructive or central in infants is considered optional, and it is based on the same criteria applied for children and adults, i.e., snoring during the event and/or increased inspiratory flattening of the flow signal and/or thoracoabdominal paradox not present during the pre-event breathing [26]. Given that paradoxical breathing is very common during REM sleep in infants, and that instead of snoring they usually manifest continuous or intermittent soft noisy breathing which cannot be reliably recorded, the distinction between central and obstructive hypopneas can be difficult if not impossible. Chacko et al., have recently proposed criteria for recognizing obstructive hypopneas in infants and in children with Spinal Muscular Atrophy [27].
(ii) In otherwise healthy children older than 1 year, hypopneas are uncommon, but may co-exist with obstructive and mixed apneas in subjects with OSAS, resolving after adenotonsillectomy if adenotonsillar hypertrophy is the predominant mechanism of upper airway obstruction [28,29]. Hence, calculation of the total AHI without classification of hypopneas into obstructive and central categories is adequate for the management of children with OSAS and without control of breathing disorders [29]. Nevertheless, the scoring of obstructive AHI in infants may indeed incorporate central hypopnea events into the calculated index, thereby artifactually increasing the severity of OSAS, and complicating recommendations for treatment. As shown by Daftary et al., in healthy neonates hypopneas are responsible for 50% of the total AHI [9].

For example, a recent systematic review of 71 studies assessing severity of upper airway obstruction in infants with Pierre Robin sequence, AHI and not obstructive AHI was the most commonly measure used, and a cut-off threshold of 20 episodes/h was applied as an indication for surgical intervention [30]. In some studies, hypopneas were not scored and a mixed-obstructive apnea index of 3 episodes/h or higher was considered an indication for insertion of a pre-epiglottic button plate, while other authors offered treatment interventions using oximetry parameters calculated from PSG. In contrast, indications for treatment are better defined in children 2–18 years old [31]. More specifically, adenotonsillectomy is more efficacious when offered to patients with moderate-to-severe disease OSAS (AHI > 5 episodes/h or oxygen desaturation (≥3%) index (ODI3) ≥ 3.5 episodes/h) compared to those with mild disease [32,33]. Thus, improved refinements in the scoring and delineation of OSAS severity categories among infants are needed, and should be predicated on standardized agreement of scoring procedures and improved understanding of the relationships between OSAS severity and downstream morbidities.

## 4. Polysomnography and Polygraphy Reference Values in the First Year of Life

Daftary et al., studied 30 otherwise healthy full-term newborns by PSG with a mean total sleep time of 260 min and mean proportions of NREM 43.3%, REM 40.6%, and transitional sleep 16.1% [9]. The median obstructive apnea index was 1.8 episodes/h (range 0.2–12.5), median mixed apnea index was 0.9 episodes/h (range 0–8.3), and median hypopnea index was 5.9 episodes/h (0.7–12.9). Kanack et al., assessed 22 healthy full-term neonates using nap PSG with a median total sleep time of 260 min and a median proportion of REM sleep 49.3% [23]. AHI, obstructive AHI, and central apnea index (CAI) for the two studies are summarized in Table 1. Of note, median and maximum AHI values in the study by Daftary et al., were almost double compared to those neonates recruited in the report by Kanack et al., thus emphasizing the clinical difficulty to define with certainty what is normal in neonatal PSG.

As expected, lower values were found in two studies performed in older infants. Brockmann et al., have presented respiratory parameters calculated from overnight PSG recordings in a limited number of young infants at the ages of 1 and 3 months [10]. Additionally, Vezina et al., have reported reference values for nocturnal polygraphy recordings performed at home in over 500 healthy children with age of approximately 1 year [22]. Limitations of this latter study were the inclusion of a minority of subjects with symptoms indicative of sleep-disordered breathing and the requirement for 50% airflow decrease in the definition of hypopnea instead of the recommended 30% [26].

If we combine the results of the two cohort studies by Brockmann et al., and Vezina et al., that were based on polygraphy without EEG, we note that obstructive apnea index reference cut-off value drops from 5.1 episodes/h (95th percentile) at 1 month of age, to 2.2 episodes/h (95th percentile) at 3 months and to 0.5 episodes/h (90th percentile) at 1 year. CAI upper reference value remains high at ages 1 and 3 months and declines by approximately 75% at 1 year (Table 1). Hypopnea index upper reference value, incorporating both central and obstructive events is unrelated to age: 3.5 episodes/h (95th percentile) at 1 month of age, 0.7 episodes/h (95th percentile) at 3 months, and 3.5 episodes/h (90th percentile) at 1 year [10,22]. Reference values for AHI, obstructive AHI, and CAI provided in the three cohort studies are summarized in Table 1.

In summary, there are currently published studies that provide reference values for the interpretation of polygraphy but not PSG respiratory parameters in infants aged 1–12 months using the current AASM Manual for the Scoring of Sleep and Associated Events [26].

## 5. Can Oximetry Replace Polysomnography or Polygraphy for the Assessment of Sleep and Breathing in Infancy?

Based on the available evidence, it is currently unknown which of the abnormalities related to upper airway obstruction, i.e., exaggerated negative intrathoracic pressure swings during obstructive events, associated intermittent hypoxemia and its consequences, alveolar hypoventilation, or frequent arousals has the most profound adverse impact on short-term and long-term health [30].

Given the difficulties in performing and interpreting PSG during the first year of life, nocturnal oximetry has been increasingly popular for the diagnosis and management of OSAS especially in settings with limited resources [34]. It is readily available in most medical facilities across the globe, far less time is required for its interpretation relative to PSG and polygraphy, characteristic desaturation patterns have been described corresponding to upper airway obstruction or obstructive lung disease, and intermittent hypoxemia is associated with known adverse alterations in multiple organ systems [35,36,37].

The Australasian Sleep Association is the first scientific society to publish Technical Specifications and Interpretation Guidelines for Overnight Oximetry as diagnostic tool for pediatric OSAS [38]. They recommend caution when nocturnal oximetry is used to diagnose OSAS in infants because central apneas may also be associated with desaturations, especially in neonates in whom immature control of breathing may predispose them to periodic breathing and result in clusters of desaturation events. Moreover, they state that central apnea and periodic breathing may co-exist with upper airway obstruction making it difficult to determine OSAS severity without PSG. Nevertheless, even when central apneas co-existing with obstructive events are demonstrated by PSG in an infant with upper airway obstruction, it is frequently difficult to determine the relative contributions of the obstructive and central components to the clinical manifestations, unless treatment is offered and the study is repeated [39,40,41]. Hence, inability to discriminate between obstructive and central events is not a unique limitation of nocturnal pulse oximetry.

A few studies that included limited numbers of otherwise healthy infants have assessed reference values for nocturnal oximetry parameters using motion-resistant equipment as single channel or as part of polygraphy or PSG [9,10,22,42,43]. ODI3 is highest during the newborn period as evidenced by Daftary et al., who performed PSG in neonates and by Terrill et al., who have analyzed nocturnal oximetry recordings from full-term infants undergoing nocturnal PSG at the ages of 2 weeks, as well as 3, 6, 12, and 24 months (Table 2) [9,44]. At the ages of 1–4 months, ODI3 95th percentile decreases by approximately 50% compared to the neonatal period according to the polygraphy study by Brockmann et al., and the PSG study by Terrill et al. [10,43]. Nevertheless, much higher ODI3 values for ages 1–4 months have been reported by Evans et al., who have applied nocturnal motion-resistant oximetry to study full-term infants, probably because wakeful periods have been included in the tracings (Table 2) [42]. ODI3 90th percentile decreases further at the ages of 6 and 12 months as reported in the polygraphy study by Vezina et al., and in the PSG study by Terrill et al. [22,43].

In summary, nocturnal oximetry may be used to assess severity of hypoxemia in infants with symptoms and signs of upper airway obstruction during sleep if PSG or polygraphy are not available or feasible [45]. Analysis of the recordings should be completed after detailed exclusion of periods corresponding to wakefulness or movement artifacts since the currently available reference ODI3 data originate from studies that have been based on PSG or polygraphy. Nocturnal oximetry could also be considered for evaluating response to treatment interventions.

## 6. Future Directions

In a recent issue of *Children*, Kang et al., have reviewed trends in diagnosing OSAS in pediatrics [46]. Although PSG is the gold standard method for diagnosis of OSAS in children and adolescents, much remains to be learned about what is normal and what is abnormal in respiratory parameters calculated from PSG, polygraphy, or nocturnal oximetry recordings in infants and especially those in their first 6 months of life:

(i) Maturation and consolidation of sleep is a dynamic process that evolves rapidly during infancy. Many young infants sleep preferentially during the day and hence performance of a nocturnal recording could fail to demonstrate the severity of clinically important REM-related obstructive events. For this reason, standardized rules need to be developed regarding the timing of a sleep study to ensure that adequate number of sleep cycles will be included in the recording so that any existing obstructive or central apneas and hypopneas are identified.
(ii) There is an urgent need for the development of reference values for respiratory parameters while sleeping during the first year of life. For this purpose, a large number of healthy full-term, but also otherwise healthy preterm infants need to be studied under standardized conditions. Currently available upper reference value for mixed obstructive apnea–hypopnea index ranges from 5.8 episodes/h at the age of 1 month to 3.6 episodes/h for a 1-year-old child. However, these values have been calculated without considering hypopneas accompanied by EEG arousals or alternatively investigators evaluated the normative frequency and the clinical implications of obstructive events unaccompanied by arousals. As a result, OSAS may be overdiagnosed in infants who undergo full video PSG and also when mixed obstructive apnea-hypopnea index is calculated based on the AASM Manual for the Scoring of Sleep and Associated Events.
(iii) During interpretation of PSG or polygraphy in an infant, an effort should be made to classify hypopneas into obstructive or central events, and standardized rules need to be developed for this purpose. In that way, over-rating of OSAS severity will be avoided eliminating the need for unnecessary treatment interventions.
(iv) More normative reference data regarding ODI3 values need to be collected from studies that are exclusively based on pulse oximetry since current data have been obtained from PSG or polygraphy recordings.
(v) Cohort studies of infants with OSAS or preferably randomized clinical trials are necessary to define obstructive AHI or ODI3 thresholds above which treatment interventions such as supraglottoplasty, pre-epiglottic button plate insertion, mandibular distraction osteogenesis, or midface advancement will be beneficial for relieving upper airway obstruction. Furthermore, improved delineation of AHI or ODI3 thresholds that are associated with increased risk of end-organ morbidities is critically needed.

It is more than clear that when evaluating sleep for clinical indications during the first year of life, infants are not just small children.

## Figures and Tables

**Table 1:** Comparison of reference values for apnea hypopnea index, obstructive apnea-hypopnea index, and central apnea index from various studies that recruited healthy infants and used AASM scoring rules.

Authors (Method)	Age Groups			
	<1 Month	1 Month	3 Months	12 Months
Daftary et al., (Nocturnal PSG) [9]	*AHI* 14.5/h (1–37.7)			
	*CAI* 3.3/h (0–27.2)			
Kanack et al., (Nap PSG) [23]	*AHI* 6.9/h (2–23)			
	*Obstructive AHI* 4.9/h (1.7–19.1)			
	*CAI* 0.7/h (0.0–12.4)			
Brockmann et al., (Nocturnal Polygraphy) [10]		*AHI* 7.8/h (95th percentile 25.5/h)	*AHI* 4.9/h (95th percentile 26.4/h)	
		*Obstructive AHI* 1.5/h (95th percentile 5.8/h)	*Obstructive AHI* 0.9/h (95th percentile 3.4/h)	
		*CAI* 5.5/h (95th percentile 20.5/h)	*CAI* 4.1/h (95th percentile 24.2/h)	
Vezina et al., (Nocturnal Polygraphy) [22]				*AHI* 4.2/h (90th percentile 10.7/h)
				*CAI* 2.5/h (90th percentile 7.1/h)

Results are presented as: median (range) or median (90th or 95th percentile). AHI: Apnea-Hypopnea Index. CAI: Central Apnea Index.

**Table 2:** Comparison of reference values for nocturnal oximetry parameters from various studies that recruited healthy infants.

Authors (Method)	Oxygen Desaturation (≥3%) Index (ODI3)				
	2 Weeks	1 Month	3–4 Months	6 Months	12 Months
Daftary et al., (Nocturnal PSG) [9]	*Median* 16.6/h *Range* 0.5–41/h				
Terrill et al., (Nocturnal PSG) [43]	*Median* 24/h *90th percentile* 47/h		*Median* 9/h *90th percentile* 19/h	*Median* 5/h *90th percentile* 12.5/h	*Median* 5/h *90th percentile* 11/h
Evans et al., (Nocturnal oximetry) [42]		*Mean* 23.4/h	*Mean* 13.9/h		
Brockmann et al., (Nocturnal Polygraphy) [10]		*Median* 8.2/h *95th percentile* 25.5/h	*Median* 7.5/h *95th percentile* 24/h		
Vezina et al., (Nocturnal Polygraphy) [22]					*Median* 6.7/h *90th percentile* 15.8/h

## Data Availability

Not applicable.
